# Anticancer activity of *Curcuma aeroginosa* essential oil and its nano-formulations: cytotoxicity, apoptosis and cell migration effects

**DOI:** 10.1186/s12906-023-04261-9

**Published:** 2024-01-02

**Authors:** Pawaret Panyajai, Natsima Viriyaadhammaa, Singkome Tima, Sawitree Chiampanichayakul, Pornngarm Dejkriengkraikul, Siriporn Okonogi, Songyot Anuchapreeda

**Affiliations:** 1 Department of Medical Technology, Faculty of Associated Medical Sciences, Chiang Mai University, Chiang Mai, 50200 Thailand; 2 Cancer Research Unit of Associated Medical Sciences (AMS CRU), Faculty of Associated Medical Sciences, Chiang Mai University, Chiang Mai, 50200 Thailand; 3 Center of Excellence in Pharmaceutical Nanotechnology, Chiang Mai University, Chiang Mai, 50200 Thailand; 4 Department of Biochemistry, Faculty of Medicine, Chiang Mai University, Chiang Mai, 50200 Thailand; 5 Department of Pharmaceutical Sciences, Faculty of Pharmacy, Chiang Mai University, Chiang Mai, 50200 Thailand

**Keywords:** *Curcuma aeroginosa*, Essential oil, Nano-formulations, Anticancer, Cytotoxicity, Apoptosis, Cell migration

## Abstract

**Background and aims:**

*Curcuma aeruginosa*, commonly known as “kha-min-dam” in Thai, holds significance in Asian traditional medicine due to its potential in treating various diseases, having properties such as anti-HIV, hepatoprotective, antimicrobial and anti-androgenic activities. This study explores the anticancer activity of *C. aeruginosa* essential oil (CAEO) and its nano-formulations.

**Methods:**

CAEO obtained from hydrodistillation of *C. aeruginosa* fresh rhizomes was examined by gas chromatography mass spectroscopy. Cytotoxicity of CAEO was determined in leukaemic K562 and breast cancer MCF-7 cell lines using an MTT assay. Cell cycle analysis and cell apoptosis were determined by flow cytometry. Cell migration was studied through a wound-healing assay.

**Results:**

Benzofuran (33.20%) emerged as the major compound of CAEO, followed by Germacrene B (19.12%) and Germacrone (13.60%). Two types of CAEO loaded nano-formulations, nanoemulsion (NE) and microemulsion (ME) were developed. The average droplet sizes of NE and ME were 13.8 ± 0.2 and 21.2 ± 0.2 nm, respectively. In a comparison with other essential oils from the fresh rhizomes of potential plants from the same family (*Curcuma longa*, *Curcuma mangga* and *Zingiber officinale*) on anticancer activity against K562 and MCF-7 cell lines, CAEO exhibited the highest cytotoxicity with IC_50_ of 13.43 ± 1.09 and 20.18 ± 1.20 µg/mL, respectively. Flow cytometry analysis revealed that CAEO significantly increased cell death, evidenced from the sub-G1 populations in the cell cycle assay and triggered apoptosis. Additionally, CAEO effectively inhibited cell migration in MCF-7 cells after incubation for 12 and 24 h. The developed NE and ME formulations significantly enhanced the cytotoxicity of CAEO against K562 cells with an IC_50_ of 45.30 ± 1.49 and 41.98 ± 0.96 µg/mL, respectively.

**Conclusion:**

This study’s finding suggest that both nano-formulations, NE and ME, effectively facilitated the delivery of CAEO into cancer cells.

## Introduction

Herbs have long been used as a source of drugs in traditional medicine. Up to now, compounds from plants have been an interesting source of anticancer drugs because they have low side effects and are easily accessible and safe. Vincristine is an example of a chemotherapeutic drug from the roots of *Catharanthus roseus*. The family Zingiberaceae, or the ginger family, is a well-known plant family in South and Southeast Asia. This family of plants is widely used in Thailand in traditional medicine for several proposes, such as the relief of stomachaches and hemorrhoids, as an herbal compress in massage therapy and as a honey balm, as well as for the improvement of blood circulation and the relief of muscular pain. The rhizomes of these plants are also used in cooking as spices and seasonings.

*Curcuma aeruginosa* is a plant belonging to the Zingiberaceae family. The common name in Thai is “Waan-ma-haa-mek” or “Kha-min-dam”. The color of its fresh rhizome is bullish violet with an ellipsoid-ovate shape. It is known as pink and blue ginger. It is widely cultured in South and Southeast Asian countries, including Thailand, Malaysia, Indonesia, Laos, Vietnam, Cambodia, India and Bangladesh. It is an aromatic 30 − 40 cm high herb. *C. aeruginosa* has been used as an ingredient in Thai herbal medicinal recipes for the treatment of various diseases in many countries. There are various biological activities of *C. aeruginosa* that have been reported, such as anti-HIV [1], antioxidant [2, 3], hepatoprotective, antiplatelet-activating factor, antinociceptive and antimicrobial activities [3–5]. Rhizomes of this plant are applied for rheumatoid disorders in Bangladesh. In Thailand, *C. aeruginosa* rhizomes are used as traditional medicines for healing the gastrointestinal tract [6].

Many essential oils from plants demonstrated several biological activities, such as antimicrobial activity [7, 8], immunomodulatory activity [9, 10], anti-oxidant activity [11] and anticancer activity [12, 13]. The essential oil of *C. aeruginosa* from Malaysia was reported to be comprised of 26 components. The major constituents were found to be curzerenone (24.6%), 1.8-cineole (11%), camphor (10.6%), zedoarol (6.3%), isocurcumenol (5.8%), curcuminol (5.6%) and furanogermenone (5.5%) which were analyzed by gas chromatography (GC) and gas chromatography-mass spectrometry (GC/MS) [3, 14]. Essential oil extract of *C. aeruginosa* from Vietnam was found to contain β-pinene (21.9%), neocurdione (16.1%) and curcumol (15.2%) [15]. The major compounds of this plant in Indonesia were tropolone (18%), eucalyptol (17.9%) and curcumol (5.7%) [16], while curzerenone (59.6%) was a major component of this plant in Nepal [17]. A major component of *C. aeruginosa* from Thailand was germacrone (23.49%). It showed antioxidant activities against DPPH^●^ and OH^●^ radicals [18]. Essential oil from the rhizome of *C. aeruginosa* exhibited potent activity against *Staphylococcus aureus*, *Bacillus cerus* and *Candida albicans* [5]. Furanodiene (1 mg/mL) isolated from *C. aeruginosa* extract showed significant antiandrogenic effects by the inhibition of 5-α reductase enzyme. In addition, essential oil from *C. aeruginosa* has been used as a topical agent in Thai traditional massage.

The study of anticancer drugs has become a priority for cancer treatment. Nowadays, chemotherapy and radiotherapy treatments are popular methods for cancer patients. However, these methods can cause uncomfortable side effects and toxicity since they can affect both normal cells and cancer cells. Moreover, drug resistance can occur in some patients after treatment, leading to relapse. Numerous studies have been conducted concerning anti-cancer effects and the mechanisms of active compounds as well as crude extracts of *C. aeruginosa* rhizomes [19, 20]. However, there are limited reports about its essential oils, such as the cytotoxicity of essential oil from *C. aeruginosa* in MCF-7 cells [16].

A drug delivery system (DDS) is a new chemotherapeutic strategy for cancer treatment. Nanotechnology has the potential for developing a DDS. Various types of nanoparticles have been developed as a DDS for anticancer drugs due to unique properties that promote the delivery and retention of particles and the enhancement of their permeability. Nanoparticles can be developed at specific sizes for potent distribution and accumulation in cancer cells. Nanoparticles are characterized by self-assembly, stability, specificity, drug encapsulation and compatibility because of their material composition.

Nanoemulsion (NE) and microemulsion (ME) are nano-formulations mainly developed to enhance the oral bioavailability of essential oils through the preparation of a system of water, oil, surfactant and co-surfactant [21]. Various essential oils have been used as an internal oil phase of the oil-in-water NE and ME processes according to the ability of these nano-formulations to improve absorption of the oils through the lipid bilayer membrane of the cells in the human body. Both NE and ME can increase the bioavailability of essential oils and improve their activity at the same time. For example, the NE of ginger essential oil demonstrated enhanced antibacterial activity against various species of bacteria [22, 23]. It has been reported that the ME of essential oil from *Zingiber cassumunar* rhizome could enhance anti-inflammatory properties without cytotoxicity to normal peripheral blood mononuclear cells (PBMCs) [24]. Although *C. aeruginosa* has demonstrated various biological activities, the anticancer activity against various cancer cells was not well reported. The present study explores the anticancer activity of *C. aeruginosa* essential oil (CAEO) against two cancer cells, K562 (chronic myelogenous leukemia cell line) and MCF-7 cells (breast cancer cell line). The activity was compared with essential oils extracted from three other potential plants, namely *Curcuma longa*, *Curcuma mangga* and *Zingiber officinale*, which are in the same family of *C. aeruginosa*. These three plants are also used in Thai traditional medicinal remedies. Four types of CAEO-loaded nano-formulations were produced in this study. The anticancer activity of the developed nano-formulations was investigated.

## Materials and methods

### Essential oil extraction and compound analysis

Fresh rhizomes of *C. aeruginosa*, *C. longa*, *C. mangga* and *Z. officinale* were collected from a local farm in Chiang Mai, Thailand, in June of 2019. The essential oils were extracted from these rhizomes by hydrodistillation as previously described [25]. The oil compositions were analyzed by GC–MS using an Agilent 6890 gas chromatography device (Agilent Technologies, Santa Clara, CA, USA) coupled to an electron impact (EI; 70 eV) HP 5973 mass selective detector (Hewlett Packard,Palo Alto, CA, USA) fitted with a column (Hewlett Packard, Palo Alto, CA, USA). The conditions used for this analysis were in accordance with those previously described [26].

### GC–MS analysis

The oil compositions were analyzed by gas chromatography mass spectroscopy (GC–MS) using an Agilent 6890 gas chromatography device (Agilent Technologies, Santa Clara, CA, USA) coupled to an electron impact (EI; 70 eV) HP 5973 mass selective detector (Hewlett Packard,Palo Alto, CA, USA) fitted with a column (Hewlett Packard, Palo Alto, CA, USA) [27].

The analytical conditions were; carrier gas: helium (ca. 1.0 mL/min), injector temperature: 260 °C, oven temperature: 3 min isothermal at 100 °C (No peaks before 100 °C after first injection), then at 3ºC/min to 188 °C and then at 20 °C/min to 280 °C (3 min isothermal), and detector temperature: 280 °C. The programmed temperature Kováts retention indices (RI) were obtained by GC–MS analysis of an aliquot of the volatile oil spiked with an n-alkanes mixture containing each homologue from *n*-C11 to *n*-C27. The identification of the compounds was based on a comparison of their mass spectra database (WILEY&NIST) and spectroscopic data. The percentage amount of each component was calculated based on the total area of all peaks obtained from the oil. The data obtained were used as a standard for further batches of the oil.

### Preparation of CAEO loaded nano-formulation

NE containing 10% CAEO (NE-CA) was prepared according to the method previously described [28] with some modification and using 25% Tween 80 as a surfactant. The mixture composed of CAEO, surfactant, and water was subjected to Ultra-Turrax T25 (Janke and Kunkel GmbH, Staufen, Germany) at a high-speed stirring rate of 12,000 rpm for 30 s to obtain a pre-emulsion. This pre-emulsion was then subjected to a high-pressure homogenizer (Micron LAB40, Homogenizer Systems, Germany) for three cycles to obtain an NE-CA. ME containing 10% CAEO (ME-CA) was prepared according to the method previously described [29] with some modification. Briefly, using 30% surfactant mixture consisting of a 2:1 ratio of Tween 80 and cosurfactant, mainly ethyl alcohol was gently mixed with CAEO to obtain 10% CAEO in the preparation. The blanks of NE-CA and ME-CA were prepared in the same manner as NE-CA and ME-CA, using water instead of CAEO. The obtained nano-formulations were characterized by size and size distribution using a photon correlation spectrophotometer (PCS) as previously described [30].

### Cell culture

K562 (RCB0027, RIKEN BioResource Research Center (BRC), Japan) and MCF-7 (HTB-22, ATCC, USA) cells were used as human cancer cell line models in this study. MCF-7 cells were cultured in DMEM (Dulbecco's Modified Eagle Medium) (Invitrogen, Carlsbad, CA, USA) supplemented with 10% fetal bovine serum (FBS) (Capricorn Scientific, Ebsdorfergrund, Germany), 100 units/mL penicillin and 100 µg/mL streptomycin (Invitrogen, Carlsbad, CA, USA). K562 cells were cultured in RPMI (Roswell Park Memorial Institute)-1640 medium (Invitrogen™, CA, USA) supplemented with 10% FBS, 2 mM L-glutamine, 100 units/mL penicillin and 100 µg/mL streptomycin (Invitrogen, Carlsbad, CA, USA). Both cancer cell lines were cultured at 37 °C in a humidified incubator with 5% CO_2_.

### MTT test

MTT (3-[4,5-dimethylthiazol-2-yl]-2,5-diphenyltetrazolium bromide) assay was performed to determine the cytotoxicity of extracts [31]. MCF-7 (5.0 × 10^3^ cells/well) and K562 (1.0 × 10^4^ cells/well) were plated in a 96-well plate and incubated at 37 °C with 5% CO_2_ overnight. Then, the cells were incubated with 0–100 µg/mL of the essential oils or 0–50 ng of CAEO/mL of the nano-formulation for 48 h. After that, MTT solution (5 mg/mL) (Sigma-Aldrich, St. Louis, MO, USA) was added and incubated for 4 h. Next, 200 µL of DMSO (Sigma-Aldrich, St. Louis, MO, USA) was added to dissolve formazan products; then, the absorbance at 578 and 630 nm were measured using a microplate reader (Metertech, Taipei, Taiwan). Four anticancer drugs, namely doxorubicin, cytarabine, cyclophosphamide and vincristine, were used as positive controls. The percentage of cell viability was calculated using the following equation:

$$\mathrm{Cell \,viability }\left(\%\right)= \left(\mathrm{At}/\mathrm{Ac}\right) \times 100;$$

where “At” is the mean absorbance in the test well and “Ac” is the mean absorbance in the vehicle control well. The average percentage of cell viability at each concentration obtained from triplicate experiments was plotted as a dose-response curve. IC_50_ was determined as the concentration of the test sample that inhibited cell growth by 50% compared with the untreated control.

### Trypan blue exclusion test

This test was performed to confirm the cytotoxicity of the test samples. K562 cells were collected and washed with ice-cold PBS, pH 7.4, three times after being incubated with 7, 9 and 13 μg/mL of CAEO. Doxorubicin (0.8 µg/mL) was used as a positive control. Then, the cells were resuspended with PBS, pH 7.4. The cell suspensions were diluted with PBS, pH 7.4, at the appropriate dilution before being mixed with 0.2% trypan blue solution at 1:2 dilution for cell count on a hemocytometer. The viable cells and the dead cells could be observed using a microscope. Viable cells show a clear cytoplasm because they can exclude trypan blue, whereas dead cells show a blue cytoplasm since they cannot.

### Cell cycle analysis

An experiment was performed to determine the effects of CAEO on cell cycle arrest and cell death [31]. K562 cells were selected as a cancer cell model. The cells were incubated with 7, 9 and 13 µg/mL of CAEO for 48 h as these concentrations were the IC_20_, IC_30_ and IC_50_ of the oil, respectively. Doxorubicin (0.8 µg/mL) was used as a positive control. After that, the cells were collected and washed with PBS, pH 7.4, three times. Then, the cells were fixed with 70% ethanol in PBS, pH 7.4, and incubated on ice for 30 min. Next, the cells were washed with PBS, pH 7.4, two times and stained with propidium iodide (PI) solution (0.02 mg/mL PI in PBS, pH 7.4). The rates of cell death and cell cycle distribution were analyzed using a flow cytometer (Cytomics FC500, Beckman Coulter, Pasadena, CA, USA).

### Apoptosis assay

To determine the apoptotic effects of CAEO in cancer cells, K562 cells (1 × 10^5^ cells/mL) were incubated with CAEO at the IC_20_, IC_30_ and IC_50_ (10, 15, 20 and 30 µg/mL, respectively) for 48 h. Doxorubicin (0.8 µg/mL) was used as a positive control. Then, cells were stained using Biolegend™ FITC Annexin V Apoptosis Detection Kit with PI (BioLegend, San Diego, CA, USA) according to the manufacturer’s instructions. In brief, cells were harvested and washed with PBS, pH 7.4, two times. After washing, cells were resuspended with binding buffer (100 µL). After that, cells were stained with Annexin V-FITC and PI for 15 min in the dark at room temperature. Next, binding buffer (400 µL) was added to each sample. The percentages of cell populations were analyzed using a flow cytometer (Cytomics FC500, Beckman Coulter, Pasadena, CA, USA) [32].

### Wound-healing assay

To determine the effects of CAEO on cell migration in cancer cells, the experiment was performed in accordance with the previously described study [33]. Briefly, MCF-7 cells (1 × 10^5^ cells/mL) were seeded into 24-well plates and incubated in a CO_2_ incubator. After cells formed a confluent monolayer, the medium was removed and cells were starved with DMEM containing 2% FBS for 18 h to suppress cell proliferation. A cell-free area was made by SPL™ Scar Scratcher (SPL Life Sciences, Gyeonggi-do, Korea); then, cells were incubated with a non-cytotoxic dose of CAEO in DMEM containing 2% FBS. Cell migration was monitored and observed under a Leica DM IL LED inverted microscope (Leica, Wetzlar, Germany) and the images of the cell-free area were captured at 0, 6, 12 and 24 h after scratching. The cell-free area was measured using ImageJ software with a wound healing size tool [34]. The percentage of wound closure was calculated by the following equation:

$$\mathrm{Wound \,closure }\left(\%\right)= \frac{\left({\mathrm{A}}_{\mathrm{t}=0\mathrm{h}}-{\mathrm{A}}_{\mathrm{t}={\varvec{\Delta}}\mathrm{h}}\right) \times 100}{{\mathrm{A}}_{\mathrm{t}=0\mathrm{h}}};$$

where “A_t=**Δ**h_” is the cell-free area measured **Δ** hours after the scratch was performed while “A_t=0 h_” is the cell-free area measured immediately after scratching (t = 0 h).

### Statistical analysis

All experiments were performed in triplicate. The average of triplicate experiments and standard deviation (SD) were used for quantification. The levels of cell populations were compared with the vehicle control in each experiment. The results are shown as mean ± SD. SPSS statistical software Ver. 22 (SPSS Inc., USA) was used for statistical analysis. Differences between the means of each sample were analyzed by one-way analysis of variance (one-way ANOVA), followed by LSD post-hoc analysis. Statistical significance was considered at *p* < 0.05 and *p* < 0.001.

## Results

### Yield and chemical analysis of CAEO

After being subjected to hydrodistillation for 3 h, 0.25 ± 0.12% oil was obtained from the fresh rhizomes of *C. aeruginosa*; the same amount obtained from the other three plants (Table 1). The outer appearance of CAEO and the other three oils was a clear and colorless oil with a distinct and pleasant odor.

**Table 1:** Percentage yield of essential oils from the family Zingiberaceae

Plant essential oil	Yield (%)
*C. aeruginosa*	0.25 ± 0.12
*C. longa*	0.78 ± 0.09
*C. mangga*	0.25 ± 0.12
*Z. officinale*	0.28 ± 0.07

Data represent mean ± SD

Chemical constituents of the extracted CAEO analyzed by GC-MS showed 16 identified compounds representing 98.02% of the total oil, including mainly Benzofuran, 6-ethenyl-4,5,6,7-tetrahydro-3,6-dimethyl-5-isopropenyl-, trans- (33.20%), germacrene B (19.12%) and germacrone (13.60%) (Table 2).

**Table 2:** Main compounds of *C. aeruginosa* essential oil analyzed by GC-MS

Retention time (min)	Compound	Peak area	Quality
14.09	Bicyclo[2.2.1]heptan-2-one, 1,7,7-trimethyl-, (1R)-	4.49	98
14.75	Isoborneol	0.97	91
22.54	Alpha-terpene	0.50	94
24.86	Beta-elemene	4.1818	95
26.56	Gamma-elemene	3.27	94
27.28	Alpha-humulene	0.96	97
28.42	Beta-cubebene	1.19	99
29.18	Benzofuran, 6-ethenyl-4,5,6,7-tetrahydro-3,6-dimethyl-5-isopropenyl-, trans-	33.20	98
29.37	Beta-elemene	1.11	90
31.36	Germacrene B	19.12	99
33.40	Epicurzerenone	2.41	87
34.31	1,H-Dimethyl-7(1-hydroxy-1-methylethyl)[3,3a,4,5,6,7]hexahydro azulene	0.57	35
35.70	6-Ethyl-1,3-dimethylindan-5-carbaldehyde	0.48	22
36.47	Furanodiene	13.29	89
36.58	Germacrone	13.60	98
39.72	8-Methyl-1-oxaspiro(4,5)decaq-3,7-dien-2-one	0.68	62

### Preparation of CAEO-loaded nano-formulation

In this study, two types of CAEO-loaded nano-formulations, NE-CA and ME-CA, were successfully prepared. Particle characterization using PCS revealed that all the CAEO droplets in the obtained nano-formulations were within the nanoscale range (Table 3). Moreover, the size distribution, expressed as polydispersity index (PDI) of the nanoparticles obtained in each formulation, was within the accepted range (0.1–0.3) for pharmaceutical use. The zeta potentials of the NE-CA and ME-CA were near zero because of the non-ionic surfactant (Tween 80) used in the systems.

**Table 3:** Size, size distribution, and zetapotential of CAEO-loaded nano-formulations

Nanoformulations	Size (nm)	PDI	Zeta potential (mV)
NE-CA	13.8 ± 0.2	0.152 ± 0.045	− 4.44 ± 0.92
ME-CA	21.2 ± 0.2	0.267 ± 0.013	− 7.57 ± 0.32

Data represent mean ± standard deviation (SD)

### Cytotoxicity of CAEO by MTT assay

The results showed that each essential oil had different cytotoxicity against cancer cells with a different IC_50_ value (Table 4). Furthermore, the results also clearly showed that CAEO had significantly higher cytotoxicity against both cells than the three other extracts. Specifically, CAEO showed significantly higher anticancer activity against both cancer cells and demonstrated the best cytotoxicity against K562 with IC_50_ of 13.43 ± 1.09 µg/mL and MCF-7 with IC_50_ of 20.18 ± 1.20 µg/mL.

**Table 4:** Cytotoxicity of plant essential oils and drugs on cancer cell lines by MTT test

Plant essential oil and drug	IC_50_	
	**K562**	**MCF-7**
*C. aeruginosa* (μg/mL)	13.43 ± 1.09	20.18 ± 1.20
*C. longa* (μg/mL)	24.07 ± 3.08	40.36 ± 4.15
*C. mangga* (μg/mL)	31.48 ± 2.23	34.01 ± 0.95
*Z. officinale* (μg/mL)	17.39 ± 1.43	24.64 ± 4.61
Doxorubicin (μg/mL)	178.67 ± 16.14	0.28 ± 0.13
Cytarabine (μg/mL)	> 100	1.87 ± 0.34
Cyclophosphamide (μg/mL)	> 400	> 400
Vincristine (μg/mL)	34.05 ± 2.48	27.40 ± 1.24

Data represent mean ± SD

### Effects of CAEO on total cell number and cell cycle

**Fig. 1:**
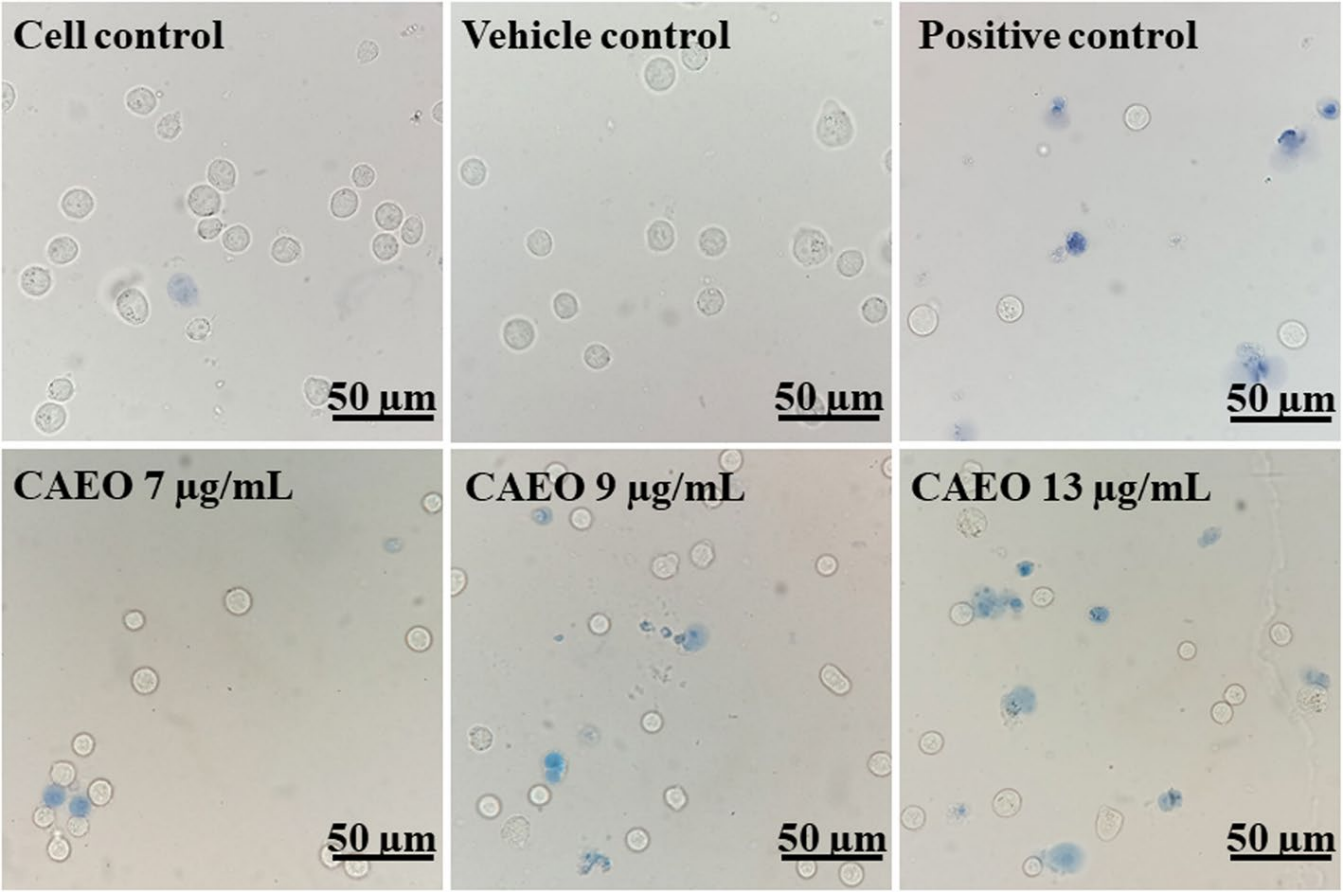
Images (40 ×) of K562 cells after incubation with CAEO or positive controls (0.8 µg/mL doxorubicin). Viable cells show clear cytoplasm and dead cells show blue cytoplasm

**Fig. 2:**
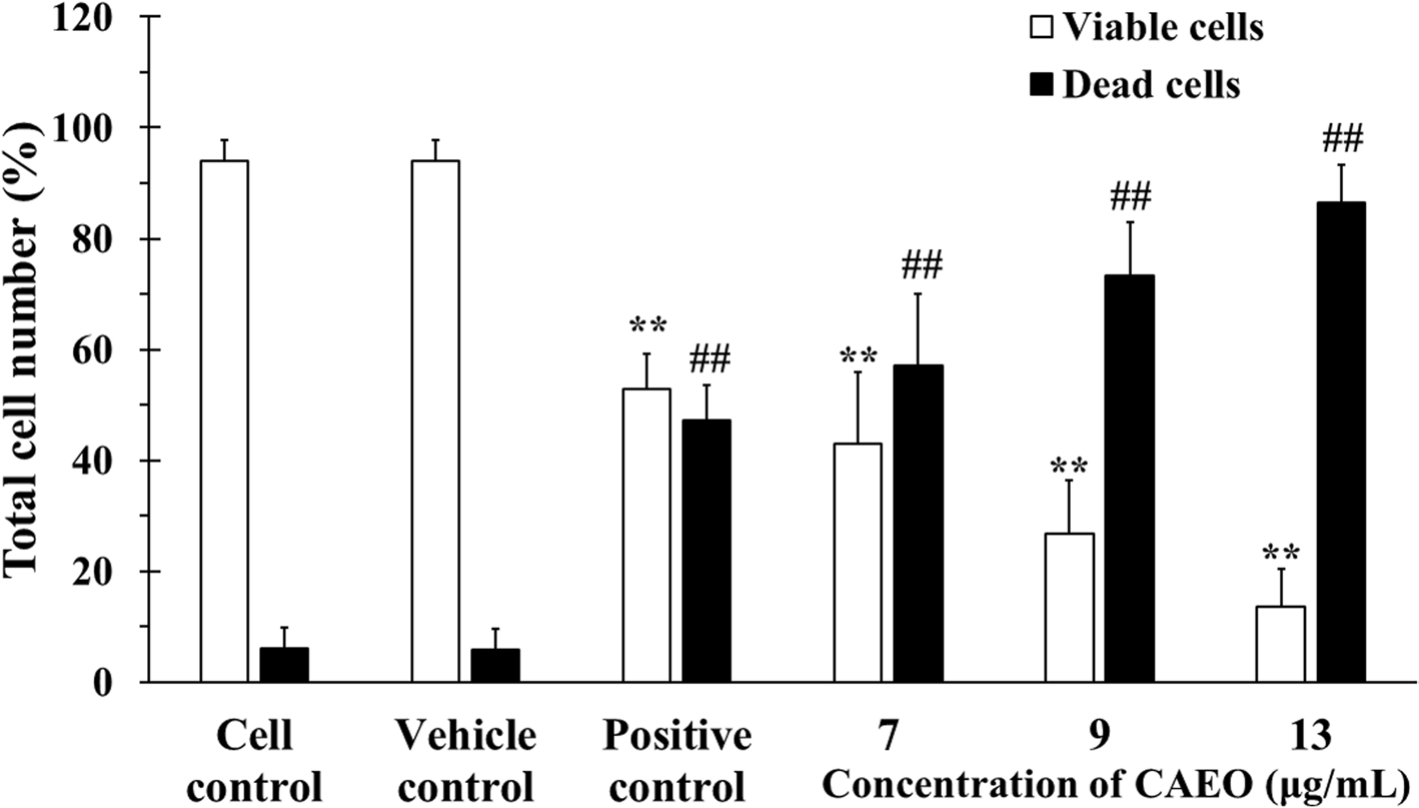
Total number of viable and dead cells after incubation for 48 h in K562 cells, including cell control (CC), vehicle control (VC) and CAEO compared with the positive control (doxorubicin at 0.8 µg/mL). Each bar represents mean ± SD of three independent experiments performed in triplicate. Asterisk (*) denotes significant differences from viable cells in vehicle control; ** *p* < 0.001. Hash (#) denotes significant differences from dead cells in vehicle control; ## *p* < 0.001. The dataset is available in S5 Dataset

The cytotoxicity of CAEO on cancer cells was confirmed using trypan blue exclusion assay and flow cytometry. K562 was used as a cancer cell model and doxorubicin (0.8 µg/mL) was used as a positive control. The viable cells were not stained and showed a clear cytoplasm whereas the dead cells showed a blue cytoplasm because they could not exclude the dye. In this experiment, a relatively high number of blue cells could be observed in the images of the cells after exposure to the positive control and increased concentration of CAEO when compared with cell and vehicle controls (Fig. 1). To confirm the number of dead and viable cells, the cells were counted using a hemocytometer. The results showed that CAEO could induce cell death after increasing the concentration of CAEO (Fig. 2).

**Fig. 3:**
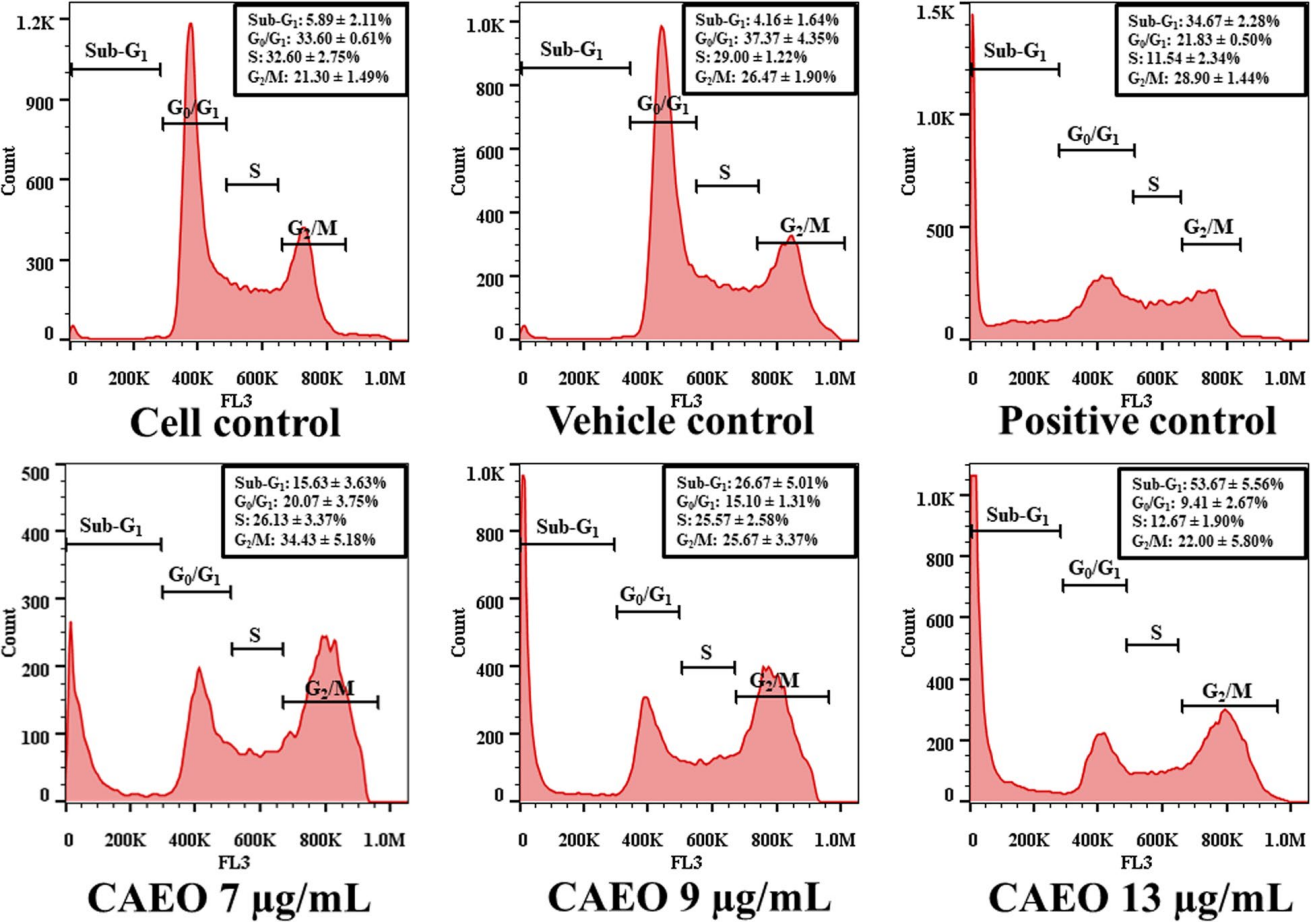
Cell cycle distribution of K562 cells after incubation with CAEO and positive control for 48 h and comparison with the cell and vehicle controls

**Fig. 4:**
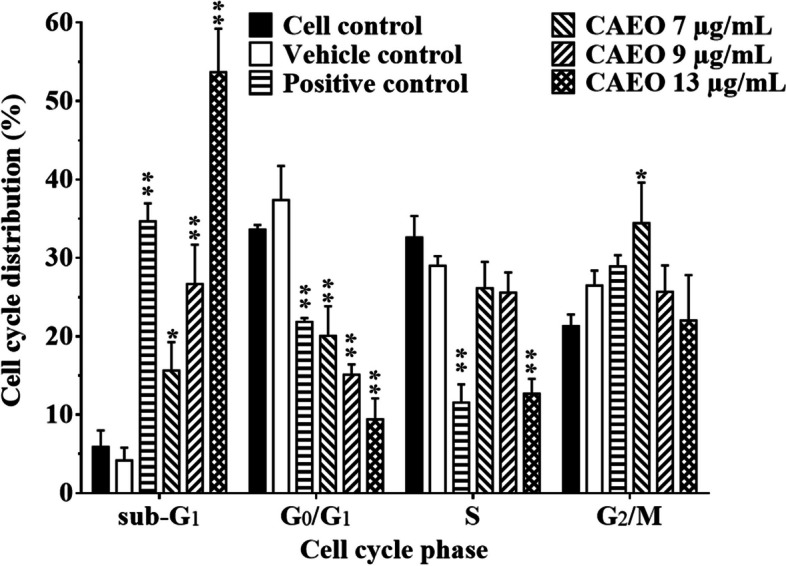
Each bar graph shows the cell cycle phases of K562 cells after incubation with CAEO and positive control for 48 h in comparison with the cell and vehicle controls. Each bar represents mean ± SD of three independent experiments performed in triplicate. Asterisk (*) denotes significant differences from vehicle control; *p < 0.05; **p < 0.001. The dataset is available in S6 Dataset

Further investigation of the anticancer activity of CAEO was done by incubating K562 cells with CAEO and analysis by flow cytometry. The sub-G_1_ population in cell cycle distribution indicating dead cells was examined. The result demonstrated an increase of sub-G_1_ peaks (Fig. 3). After cell populations in each phase of the cell cycle were analyzed, the results demonstrated that CAEO had anticancer activity and induced cell death when compared with the cell control and the vehicle control. Additionally, with CAEO at 7, 9 and 13 µg/mL, the sub-G_1_ population increased in a dose-dependent manner (Fig. 4). Furthermore, G_2_/M population was increased after treatment with a non-cytotoxic dose of CAEO (7 µg/mL) for 48 h.

### Effect of CAEO on cell apoptosis

**Fig. 5:**
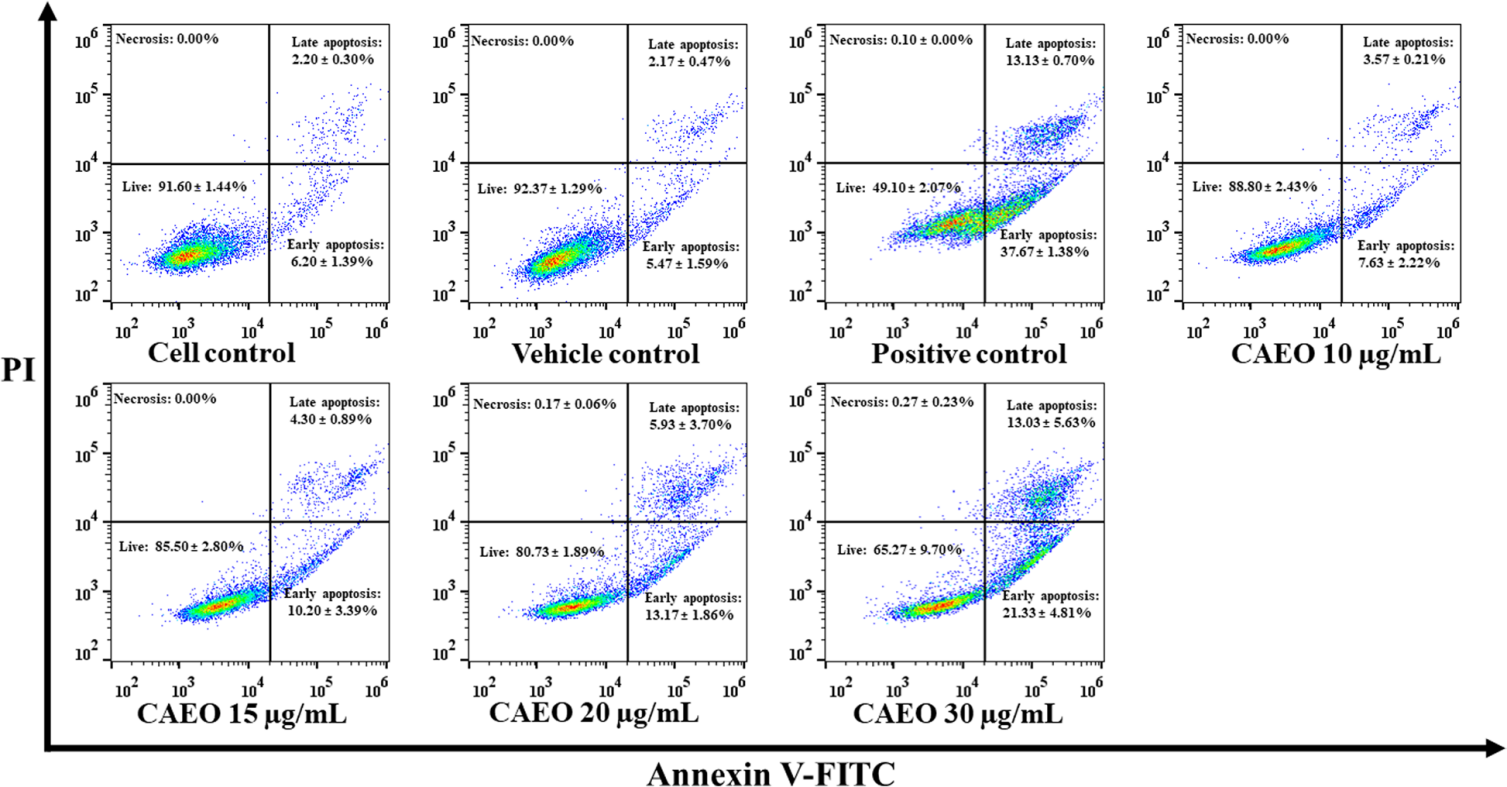
Apoptosis assay by flow cytometry after staining with annexin V-FITC/propidium iodide (PI). K562 cells were incubated with CAEO at the concentrations of 10, 15, 20 and 30 µg/mL. Representative flow cytometry dot plot indicating the cell population in apoptotic and necrotic quadrants after treatment

**Fig. 6:**
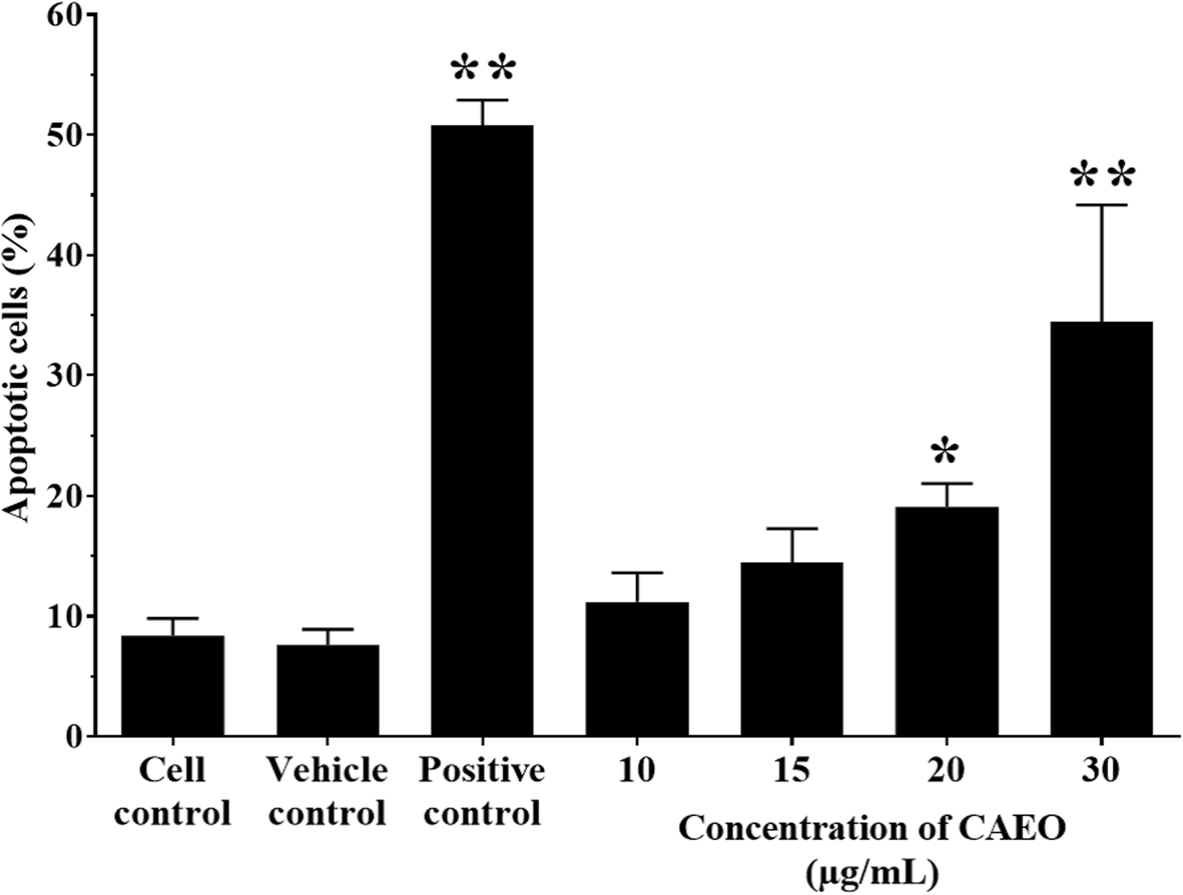
Each bar graph shows apoptotic cells (%) by flow cytometry after incubation with CAEO. K562 cells were treated with CAEO at the concentrations of 10, 15, 20 and 30 µg/mL and stained with annexin V-FITC/propidium iodide (PI). The percentage of apoptotic cells in each treatment was statistically compared with cell and vehicle controls. Each bar represents mean ± SD of three independent experiments performed in triplicate. Asterisk (*) denotes significant differences from vehicle control; *p < 0.05, **p < 0.001. The dataset is available in S7 Dataset

This study was performed to confirm the effect of CAEO on cell apoptosis in K562 cells that appeared at the sub-G1 phase in cell cycle distribution. The percentage of apoptotic cells was measured after incubation with CAEO at the concentration of 10, 15, 20 and 30 µg/mL. Doxorubicin (0.8 µg/mL) was used as a positive control (50.80 ± 2.07%). The result showed that CAEO at a concentration of 20 and 30 µg/mL significantly increased apoptotic cell population (19.10 ± 1.95 and 34.47 ± 9.71%, *p* < 0.05) when compared with cell control and vehicle control (8.40 ± 1.44% and 7.63 ± 1.29%, respectively) (Figs. 5 and 6).

### Effect of CAEO on cell migration

**Fig. 7:**
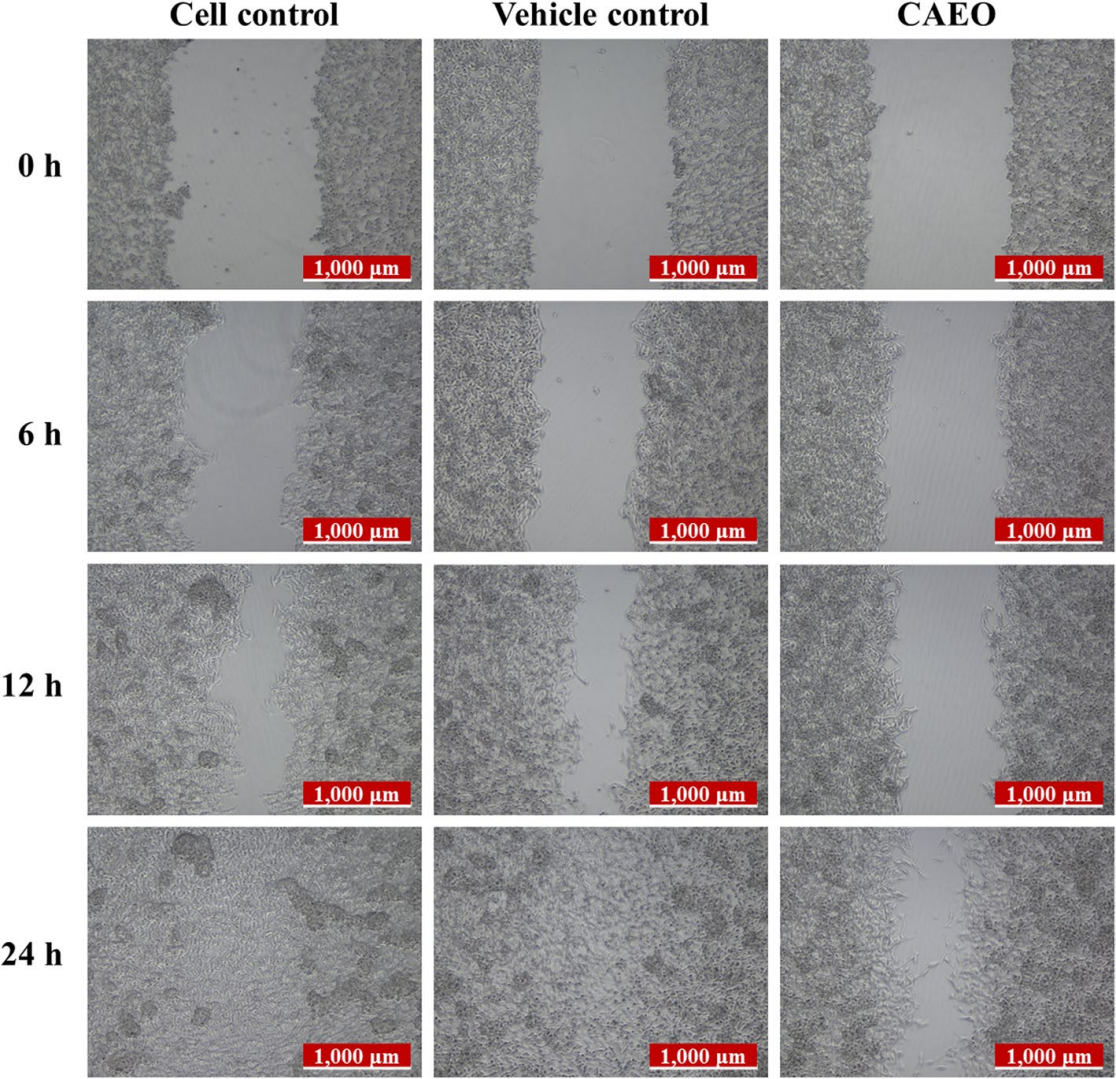
Representative images (10 ×) of wound area of MCF-7 cells after incubation with CAEO at IC_20_ concentration. Representative images show the anti-migration effect of CAEO on MCF-7 cells at 12 and 24 h

**Fig. 8:**
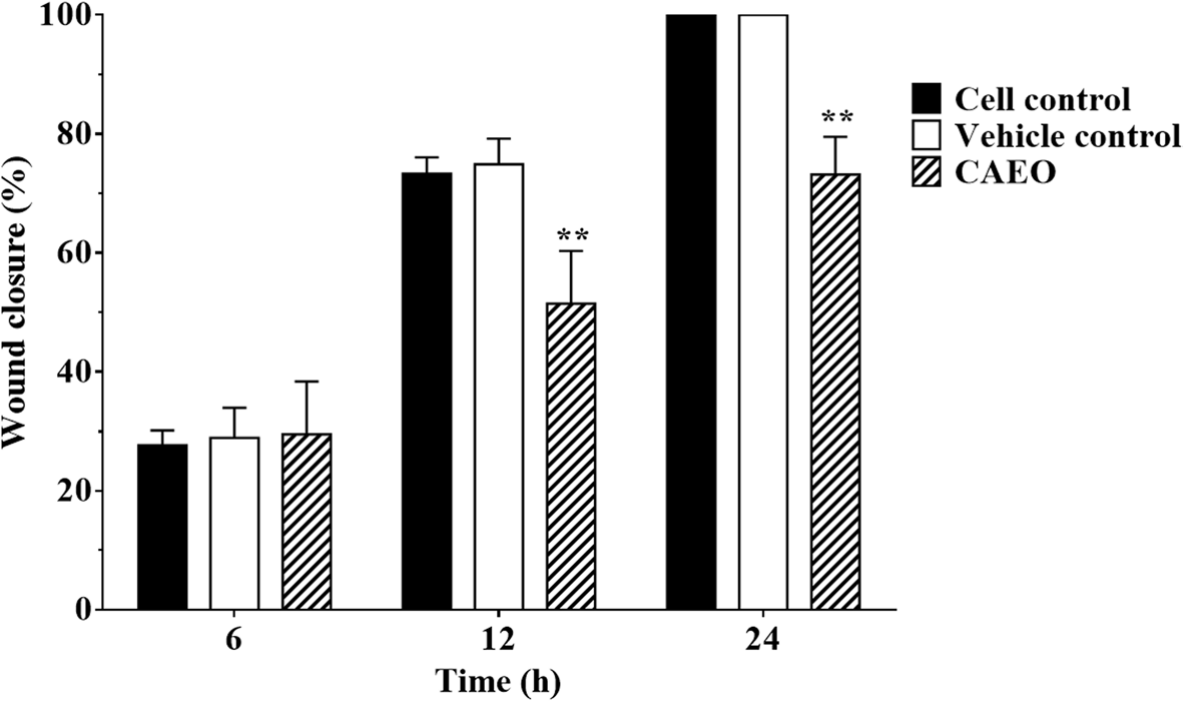
Each bar graph shows the wound closure area (%) of MCF-7 in each treatment. MCF-7 cells were incubated with CAEO at IC_20_ concentrations for 6, 12 and 24 h. Wound areas were measured at each time point and expressed as a percentage of reduction area in comparison with 0 h of incubation. The percentages of wound closure were statistically compared. Each bar represents mean ± SD of three independent experiments performed in triplicate. Asterisk (*) denotes significant differences from vehicle control; ** *p* < 0.001

To investigate the effect of CAEO on cell migration, MCF-7 cells were used as the model of cancer cell migration and investigated by wound-healing assay. In this study, after incubation for 24 h, the result indicated that cell-free areas of vehicle and cell controls were decreased and eventually closed, while CAEO treatment still had a gap (Fig. 7). Then, wound closure was calculated and analyzed from the cell-free area. The result demonstrated that CAEO significantly inhibited the migration of MCF-7 cells when compared with both controls after incubation for 12 and 24 h with wound closure of 51.47 ± 8.84 and 73.15 ± 6.38%, respectively (Fig. 8).

### Cytotoxicity of CAEO loaded nanoformulations

The developed CAEO loaded nanoformulations were investigated for their anticancer activity, in comparison with CAEO alone, against two cancer cells (K562 and MCF-7 cells) using MTT assay. The results demonstrated that the IC_50_ of CAEO alone (13.43 ± 1.09 µg/mL) was significantly less than that of NE-CA and ME-CA (45.30 ± 1.49 and 41.98 ± 0.96 μg/mL, respectively) (Table 5).

**Table 5:** Cytotoxicity of CAEO loaded nano-formulations on cancer cell lines

Nanoformulations	IC_50_ value (μg of essential oil/mL)	
	**K562**	**MCF-7**
NE-CA	45.30 ± 1.49	18.87 ± 5.77
NE-CA blank	401.34 ± 33.47	118.92 ± 40.45
ME-CA	41.98 ± 0.96	36.81 ± 3.32
ME-CA blank	456.34 ± 18.04	388.74 ± 32.58

Data represent mean ± standard deviation (SD)

## Discussion

*C. aeruginosa* can be found in traditional drug recipes in South and Southeast Asia. The essential oil compositions of this plant have been reported from several countries, i.e. Malaysia [3, 14], Thailand [18], Indonesia [16], and Nepal [17]. In this study, the main compound of interest from CAEO was a benzofuran; however, the previous study reported that major compounds of CAEO were tropolone (18.1%), eucalyptol (17.9%) and curcumol (5.7%) [16]. Compared with the previous reports, the compositions of CAEO obtained from the present study were slightly different. Generally, the composition profile, concentration of individual components, and yield of the essential oils of one plant can be different depending on intrinsic factors, such as plant cultivars [35–37] and extrinsic factors, i.e. environmental factors, cultivation conditions, geographical location and harvest duration [38–43].

To investigate the anti-cancer activity of CAEO in the current study, CAEO was allowed to be in contact with K562 and MCF-7 cancer cell lines and the cytotoxicity was compared with three other essential oils using MTT assay. The result showed that all four essential oils possessed better cytotoxicity against K562 cells than MCF-7 cells. Interestingly, CAEO had significantly higher cytotoxicity against both cells than the other three plant extracts. Comparing with the chemotherapeutic drugs used as positive controls, CAEO showed higher effective anticancer activity than cytarabine and cyclophosphamide against K562 cells and cyclophosphamide against MCF-7 cells. Previously, it was reported that CAEO decreased the cell viability of MCF-7 cells at an increasing concentration of CAEO by MTT assay; however, the previous result showed that the IC_50_ value of CAEO in MCF-7 cells was eight times higher than the value obtained in this study [16].

Moreover, trypan blue exclusion assay and flow cytometry were used to confirm the cytotoxicity of CAEO. The result showed that an increasing number of dead cells could be observed under light microscope and cell cycle analysis (at sub-G1 phase) after treatment with the positive control (0.8 µg/mL doxorubicin). and increasing concentrations of CAEO. These results indicated that CAEO could induce dead cells in a dose-dependent manner. Moreover, a non-cytotoxic dose of CAEO also increased cell population at G_2_/M phase, as observed by flow cytometry. Interestingly, this is the first time it has been reported that the essential oil from the rhizome of *C. aeruginosa* could induce cell apoptosis in a cancer cell line. Previously, there had been a report that methanolic extract from the rhizome of this plant possessed good cytotoxicity and induced cell apoptosis in A549 (lung carcinoma) and HeLa (cervical cancer) cells through caspase-dependent pathways [20]. Moreover, there is benzofuran, 6-ethenyl-4,5,6,7-tetrahydro-3,6-dimethyl-5-isopropenyl-, trans- or curzerene, which is the main compound in CAEO that could induce cell apoptosis in glioblastoma cell lines by inhibiting the mTOR pathway and downregulating GSTA4 [44]. Meanwhile, there was no difference in the percentage of apoptotic cells when comparing CAEO at concentrations of 10 and 15 µg/mL with those of cell control and vehicle control. However, increasing CAEO concentration induced cell apoptosis in a dose-dependent manner. Thus, these results indicated that CAEO is capable of inducing cell cycle arrest and cell apoptosis.

Furthermore, there was a report that comosone II from the rhizome of this plant had anti-migration activity in MDA-MB-231 cells (breast cancer) in a dose-dependent manner [19]. A previous study also showed that curzerene could inhibit the migration and invasion of glioblastoma cell lines by downregulating MMP9 and EMT [44]. However, there is no report about the anti-migration activity of essential oils from *C. aeruginosa*. In this study, CAEO could significantly inhibit cell migration after 12–24 h when compared with cell control and vehicle control. Thus, this result showed that CAEO had anti-migration activity and this is the first time it has been reported that CAEO could inhibit cell migration in cancer cells.

Nanotechnology can be applied in medical and pharmaceutical fields to make advances in diagnosis and treatment. Anticipated applications include in vitro and in vivo drug delivery [45, 46], drug solubility and stability enhancement [47, 48], and the production of improved biocompatible materials [49]. Several types of nano-formulations have been developed and had their advantages shown in the improvement of the effect of chemotherapeutic drugs [50–52]. In this study, to improve the cytotoxicity of CAEO on cancer cells, CAEO was encapsulated into nanoparticles of NE and ME. These two nanoformulations were selected to load CAEO because they possess high benefits on drug loading capacity and stability. An ME is a formulation used to incorporate plant essential oil because it is an isotropic colloidal system that is formed spontaneously from appropriate combinations of oil, water and surfactant/co-surfactant mixtures, For NE, is usually a dispersion of oil droplets in the aqueous system containing sufficient surfactant. The advantage of both ME and NE is that they possess high kinetic stability due to their extremely small droplet size of the internal phase, approximately 20–200 nm [53–55] which is below the wavelength of visible light. The essential oil loaded in these nanoformulations can act as an internal phase of the systems and active ingredient at the same time, in the case where the oil has its own effect.

The results showed that the developed nano-formulations had less cytotoxicity than CAEO alone, except NE-CA against MCF-7 cells. Meanwhile, the blank formulations had no activity. Moreover, each CAEO-loaded nano-formulations also showed different cytotoxicity on both cancer cells. Between both cancer cells, K562 cells exhibited the most sensitivity to CAEO, while MCF-7 cells were also sensitized to the produced CAEO nano-formulations, indicating a significantly higher efficiency of these nano-formulations in the delivery of CAEO into cancer cells.

## Conclusion

In this study, the anticancer potential of CAEO on two cancer cell lines, K562 and MCF-7, was investigated and compared with three other essential oils from plants in the same family. Remarkably, CAEO had higher cytotoxicity in both cancer cell lines than the three other essential oil plants. Between both cancer cell lines, CAEO shows the most pronounced cytotoxicity in K562 cells. The observed anticancer activity of CAEO displayed a dose-dependent pattern in the induction of cell death. This phenomenon was perceptible through flow cytometry, which demonstrated an augmented sub-G1 phase in the cell cycle analysis and was corroborated by apoptotic induction evident in the apoptosis assay after incubation with CAEO at 10, 15, 20 and 30 µg/mL. Furthermore, CAEO also exhibited an anti-migration effect on MCF-7 cells after incubation for 12 and 24 h. To enhance the efficacy of CAEO, nanoparticle formulations were engineered. The integration of CAEO into these nano-formulations led to a noteworthy augmentation in its anticancer efficacy. Intriguingly, between the two developed nano-formulations, ME-CA demonstrated superior anticancer activity compared with NE-CA in K562 cells, yet yielded the opposite outcome in MCF-7 cells. The amalgamation of our findings underscores the promising potential of CAEO and its nano-formulations (NE-CA and ME-CA) as potent agents in the realm of anticancer therapeutics.

## Data Availability

The datasets used and/or analyzed during the current study are available from the corresponding author by reasonable request.
